# A case of unexpected diagnosis of fibronectin glomerulopathy with histological features of membranoproliferative glomerulonephritis

**DOI:** 10.1186/s12882-024-03456-7

**Published:** 2024-01-22

**Authors:** Misa Hata, Takayasu Mori, Yurika Hirose, Yuriko Nishida, Shintaro Mandai, Fumiaki Ando, Koichiro Susa, Soichiro Iimori, Shotaro Naito, Eisei Sohara, Tatemitsu Rai, Towako Taguchi, Shohei Tomii, Kenichi Ohashi, Shinichi Uchida

**Affiliations:** 1 Department of Nephrology, Graduate School of Medical and Dental Sciences, Tokyo Medical and Dental University (TMDU), Tokyo, Japan; 2 Department of Comprehensive Pathology, Graduate School of Medical and Dental Sciences, TMDU, Tokyo, Japan; 3 Division of Surgical Pathology, Tokyo Medical and Dental University Hospital, Tokyo, Japan; 4 Department of Human Pathology, Graduate School of Medical and Dental Sciences, TMDU, Tokyo, Japan; 5 Department of Nephrology and Hypertension, Dokkyo Medical University, Tochigi, Japan

**Keywords:** Fibronectin glomerulopathy, Membranoproliferative Glomerulonephritis, Prednisolone, Nephrotic syndrome

## Abstract

Fibronectin (FN) glomerulopathy (FNG), a rare autosomal hereditary renal disease, is characterized by proteinuria resulting from the massive accumulation of FN in the glomeruli. It typically affects individuals aged 10–50 years. In this report, we describe the case of a 57-year-old man who was diagnosed with FNG through genetic analysis and histological examination that revealed membranoproliferative glomerulonephritis. Despite treatment with prednisolone, the therapeutic response was unsatisfactory. Prednisolone was subsequently tapered and discontinued because the patient had pulmonary thromboembolism. Subsequent comprehensive genetic testing, which was initially not conducted because the patient’s parents did not have a history of kidney disease, identified a known disease-causing variant in the *FN1* gene, indicating a de novo variant. FNG was further confirmed by positive staining of glomeruli with FN using an IST-4 antibody. Although corticosteroid therapy is commonly employed as the initial treatment for MPGN, its appropriateness depends on the underlying etiology. Thus, clinicians must be aware of potential rare genetic causes underlying MPGN.

**Supplementary Information:**

The online version contains supplementary material available at 10.1186/s12882-024-03456-7.

## Background

Fibronectin (FN) glomerulopathy (FNG) is an uncommon autosomal dominant kidney disease. It is characterized by proteinuria, and some patients also exhibit nephrotic syndrome. Approximately half of the patients present with hematuria and hypertension, and 25% progress to end-stage renal disease within 15–20 years of disease onset, necessitating renal replacement therapy [1]. The precise number of cases is unknown, primarily due to the rarity of the disease, with no case reports or prevalence rates documented in previous reviews. In our investigation, we conducted a basic survey using PubMed searches. Specifically, a MeSH search was performed employing the keywords "glomerulopathy with fibronectin deposits" to encompass all available reports to date. We identified a total of approximately 113 cases, including those reported in case series. FNG is caused by variants in the *FN1* gene located on chromosome 2. While some FNG cases have been reported to display membranoproliferative glomerulonephritis (MPGN)-like lesions [2], previous case reports lacked concurrent discussion of histology and genotype. Simultaneous consideration of these factors is essential for accumulating genotype–phenotype correlation data in similar cases in the future. Currently, no specific treatment is available for this disease; although previous reports [3] have mentioned the potential efficacy of steroids, their definitive role remains uncertain. In this report, we present a case of FNG with a pathological diagnosis of MPGN. The diagnosis was confirmed by retrospective genetic analysis after the initiation of steroid therapy because the patient’s parents did not have a history of kidney disease.

## Case presentation

A 57-year-old Japanese man presented to our department because of urinary protein and renal dysfunction identified during a routine medical check-up. He had a history of hematuria since his 20 s. At the age of 53, significant urinary protein (2 +) became apparent; however, it had not been thoroughly investigated. The patient had mild hypertension, with a systolic blood pressure of approximately 130 mmHg. While his parents had no history of kidney disease, his maternal grandmother had kidney disease; however, specific details were unknown. In addition, a maternal cousin had undergone kidney transplantation in her 30 s. On the initial visit, laboratory examinations revealed mild renal dysfunction (serum creatinine [s-Cr] 1.03 mg/dL), proteinuria (urinary protein creatinine ratio [UPCR] 1.21 g/gCr), and hematuria (20–29 red blood cells [RBCs]/high-power field). Serum immunological examination revealed a weakly positive PR3-ANCA level of 4.9 U/L. Serum complement levels (C3, C4, and CH50) were within the normal range (Table 1). To confirm the diagnosis, a percutaneous renal biopsy was performed.

**Table 1:** Laboratory Data from Initial Admission

**Blood tests**		**Immunoserology**	
White Blood Cell	6,900 /μL	IgG	1,480 mg/dL
Hemoglobin	11.9 g/dL	IgA	203 mg/dL
Platelet	19.5 × 10^4^ /μL	IgM	88 mg/dL
Total protein	6.3 g/dL	Complement 3	122 mg/dL
Albumin	3.6 g/dL	Complement 4	30 mg/dL
Urea nitrogen	22 mg/dL	CH50	60 U/mL
Creatinine	1.12 mg/dL	ASO	190 IU/mL
eGFR	53.7 mL/min/1.73 m^2^	ASK	× 1280
Sodium	143 mEq/L	ANA	< × 40
Potassium	4.0 mEq/L	Anti-GBM antibody	< 2.0 U/mL
Chloride	107 mEq/L	MPO-ANCA	< 1.0 U/mL
Calcium	8.9 mg/dL	PR3-ANCA	4.9 U/mL
Phosphate	3.0 mg/dL	HBs-Ag	-
Total bilirubin	0.6 mg/dL	HCV-Ab	-
AST	14 IU/L		
ALT	9 IU/L	**Urinalysis**	
LDH	167 IU/L	specific gravity	1.020
Creatinine kinase	88 IU/L	pH	6.5
C-reactive protein	0.05 mg/dL	Protein	0.82 g/gCr
		Red Blood Cell	10–19 /high power Field

*eGFR* Estimated glomerular filtration rate, *AST* Aspartate aminotransferase, *ALT* Alanine aminotransferase, *LDH* Lactate dehydrogenase, *ASO* Anti-streptolysin O antibody, *ASK* Anti-streptokinase antibody, *ANA* Antinuclear antibody, *GBM* Glomerular basement membrane, *HBs-Ag* Hepatitis B surface antigen, *HCV-Ab* Hepatitis C virus antibody; human immunodeficiency virus antibody

**Fig. 1:**
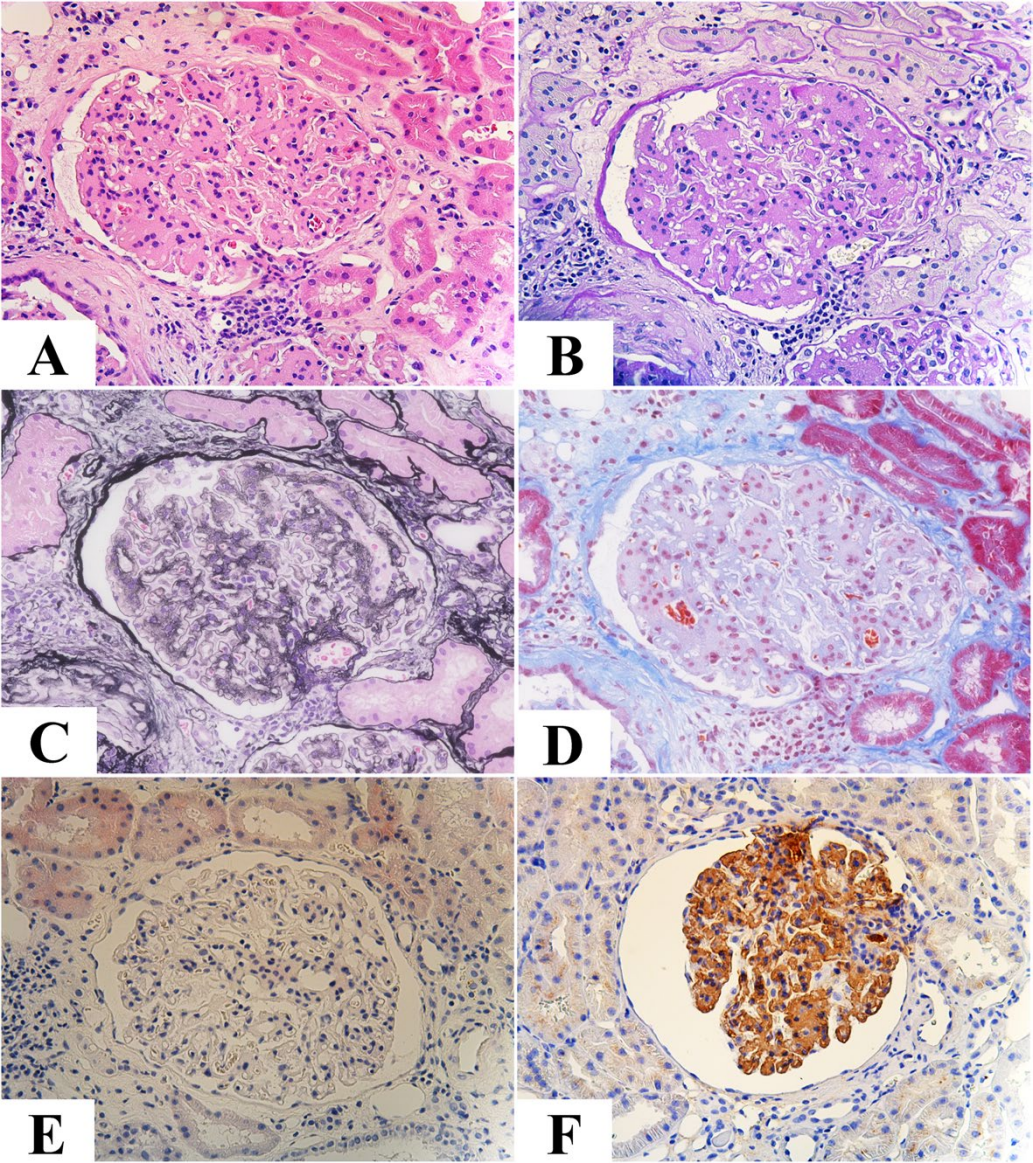
Light and electron microscopic examination. **A** and **B** Diffuse mesangial cell proliferation and increased mesangial matrix (HE and PAS staining, respectively, × 200). **C** PAM staining revealed thickening of the basement membrane with extensive mesangial infiltration and doubling. The lobulated segment exhibited scattered areas with methenamine silver-negative staining. (× 200). **D** MT staining showed a mild enhancement of trichrome red consistent with the lobulated region (× 200). **E** Congo-red staining yielded negative results. **F** Fibronectin (IST-4) staining exhibited a diffuse positive signal in the glomeruli, predominantly in the mesangial region (× 200). HE, hematoxylin and eosin staining; PAS, periodic acid-Schiff; PAM, periodic acid-methenamine-silver; MT, Masson trichrome staining

**Fig. 2:**
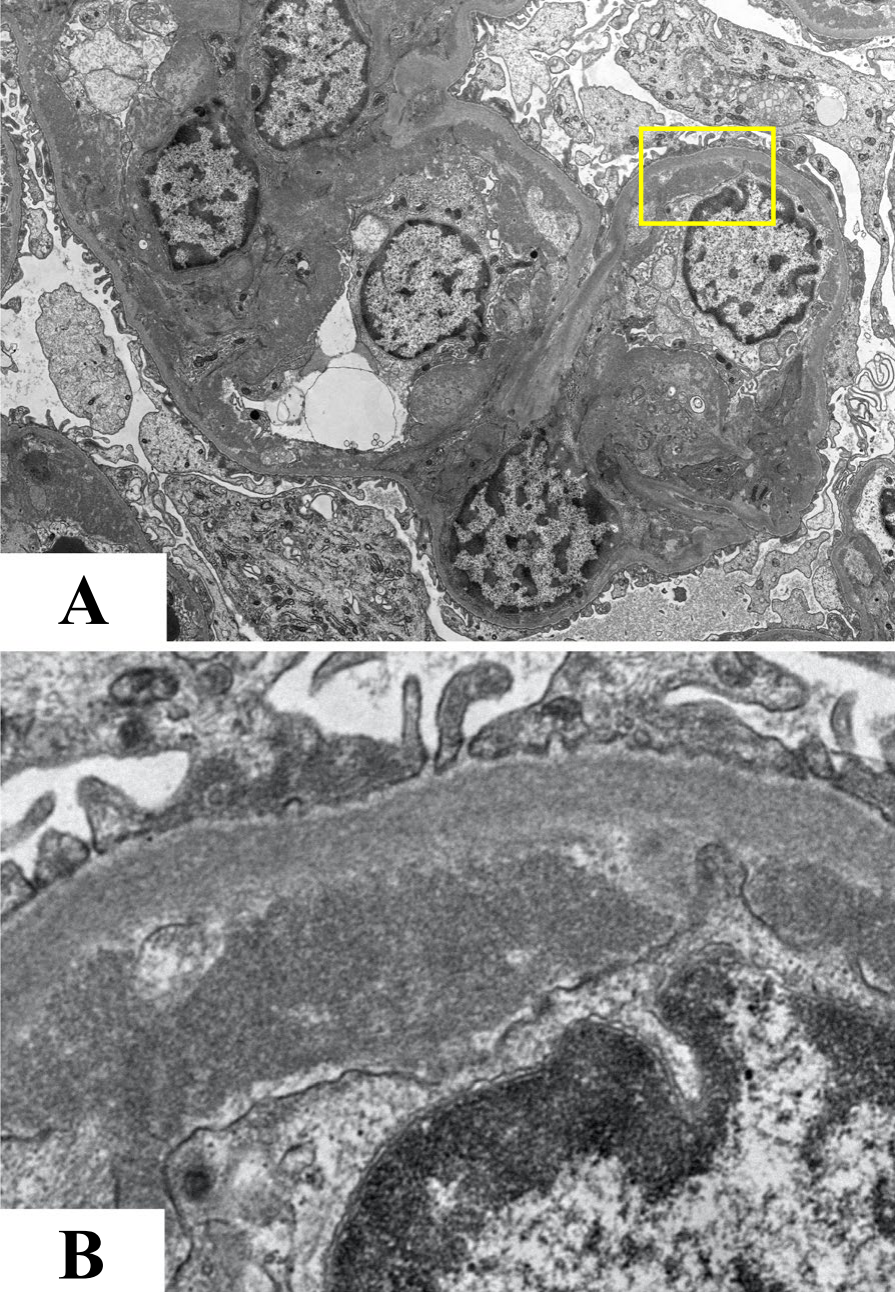
Electron microscopy (EM) of the kidney biopsy specimen. **A** High electron density deposits were found in the paramesangial area and subendothelium (EM × 1000). **B** Highly magnified image of the area encircled by the yellow square in image 2A

Figures 1, 2 and Supplementary Fig. 1 present the renal biopsy tissue. Light microscopy examination detected 30 glomeruli. Diffuse mesangial cell proliferation was observed, accompanied by thickening of the basement membrane, extensive mesangial infiltration, and doubling. Both Congo-red and Direct fast scarlet (DFS) staining, which are used to detect amyloid components, yielded negative results, and no staining bias was observed for either kappa or lambda. Immunofluorescent staining revealed mild glomerular deposition of IgG only (Supplementary Fig. 1). Furthermore, electron microscopy revealed electron-dense deposits (EDD) with high density in the paramesangial area and subendothelium, which appeared to consist of amorphous components, with less apparent fibrillary material (Fig. 2).

Following the identification of membranoproliferative pattern GN, the patient was diagnosed with idiopathic MPGN. He was readmitted to the hospital, and oral prednisolone treatment at a dose of 60 mg/day (1 mg/kg/day) was initiated. Upon admission, laboratory results indicated s-Cr level of 1.11 mg/dL, proteinuria of 0.78 g/gCr, and hematuria of 10–19 RBCs/HPF. During the hospitalization, the patient’s urinary protein levels decreased, and after 25 days, he was discharged with the prednisolone dosage reduced to 40 mg/day. However, his renal function gradually deteriorated during outpatient follow-up. During a visit to the outpatient clinic after discharge, his blood pressure dropped to 115/88 mmHg, whereas the urinary protein level remained at approximately 0.5 g/gCr. This may be partly caused by a pre-renal factor attributed to the use of diuretics (20 mg/day furosemide) to manage water retention associated with corticosteroid administration. Owing to the perceived limited therapeutic effect of prednisolone, the dose was gradually reduced to 20 mg/day. Furthermore, considering the COVID-19 pandemic at the time, there were also concerns regarding its effect.

On June 2, 2020, the patient presented to the emergency department with sudden right-sided abdominal pain. Laboratory tests revealed high D-dimer levels (67.83 μg/mL), and contrast-enhanced computed tomography (CT) confirmed the presence of thrombi in both the inferior pulmonary arteries and right inferior femoral vein. Pulmonary thromboembolism (PE) was established as a new event following the initiation of prednisolone therapy, as no evident thrombus formation was observed in a previous CT scan conducted on January 4. Following consultation with a cardiologist, apixaban therapy was initiated. Oxygen administration was gradually reduced over a few days, and the patient maintained a stable respiratory function without hypoxemia while breathing room air. During the treatment for pulmonary thromboembolism, the s-Cr levels rapidly increased to 1.3 mg/dL, and urinary protein transiently rose to 2.3 g/gCr. These changes can be partly attributed to the high blood pressure (increasing from a baseline of 120/80 mmHg to 140/100 mmHg) and high renal vein pressure caused by increased right ventricular system venous pressure. Considering that prednisolone lacked therapeutic efficacy and the presence of thromboembolism, prednisolone was further tapered and ultimately discontinued on January 4, 2021.

Given that the patient’s parents and first-degree relatives had no history of renal dysfunction or urinary abnormalities and the patient presented with MPGN without hypocomplementemia or any history suggestive of atypical hemolytic uremic syndrome (aHUS), genetic causes, including abnormalities in the complement system, was initially unlikely. Consequently, genetic testing was not performed at the outset. However, we subsequently conducted a comprehensive genetic screening using targeted next-generation sequencing (NGS) covering exons and splicing regions of 121 known genes responsible for major inherited kidney diseases (Supplementary Table) [4]. The results revealed a known in-frame deletion variant, c.4415_4417del: p.(Pro1472del), which had previously been reported as a disease-causing variant in FNG [5]. Immunostaining with FN (IST-4: Monoclonal anti-fibronectin antibody produced in mouse, clone IST-4, SIGMA-ALDRICH, F0916) demonstrated diffuse positive staining in the glomeruli, predominantly in the mesangial region, further confirming the diagnosis of FN nephropathy. The patient’s son, who had occasional episodes of microscopic haematuria during medical check-ups, also underwent genetic testing, and the same FN1 variant was identified. The co-segregation pattern provided additional support for the pathogenicity of the identified variant. After discontinuing the steroid therapy, the patient’s s-Cr level remained stable, ranging from approximately 1.3–1.4 mg/dL, and urinary protein levels were well controlled below 0.25 g/gCr with conservative treatment primarily using angiotensin II receptor blockers (ARBs). Notably, even without the use of anticoagulants, no signs of hypoxia were observed.

## Discussion and conclusions

We encountered a case of FNG in a 57-year-old male patient who exhibited MPGN pathology, which was subsequently confirmed through genetic testing. The diagnosis was substantiated by both histological examination and genetic co-segregation analysis. Notably, the patient’s parents had no recorded history of renal disease or abnormal urinalysis, suggesting a probable de novo variant. Unfortunately, due to the parents’ demise, further verification was challenging.

FN is a high-molecular-weight glycoprotein component of the extracellular matrix. It exists as a circulating dimer and exhibits binding affinity toward heparin and integrins [6]. In normal physiological conditions, FN is primarily synthesized by mesangial cells in the kidney and liver. However, the precise mechanisms underlying the pathogenesis of FN accumulation remain incompletely understood. We hypothesized the involvement of the formation of FN mutants that are not excreted or the attachment of circulating factors. The deposits primarily consist of soluble plasma-derived FN rather than the insoluble cellular form. In vitro studies have demonstrated impaired binding of this FN variant to heparin on podocytes and endothelial cell surfaces [6]. Consequently, it exhibited the ability to induce endothelial cell expansion and podocyte cytoskeletal reorganization [5]. Another proposed mechanism involves impaired FN catabolism. Furthermore, the precipitate contains high levels of fibulin 1 and 5, which can bind to and/or regulate FN.

The typical pathological findings were as follows: periodic acid-Schiff (PAS)-positive deposits in the subendothelium and mesangium, hyperplasia of mesangial cells, and glomerulomegaly by light microscopy, negative or very light staining by fluorescent antibody, and unstructured granular deposits in the subendothelium and mesangium by electron microscopy, partially surrounded by bright bundles of fine fibers. In this specific case, we initially did not consider FN as a potential differential diagnosis. This was due to the presentation of membranoproliferative pattern GN with mild IgG deposition, along with the presence of amorphous EDD in the subendothelial or paramesangial region, which we initially considered as nonspecific. However, in retrospect, these findings support the diagnosis of FN, except there was no apparent family history.

**Fig. 3:**
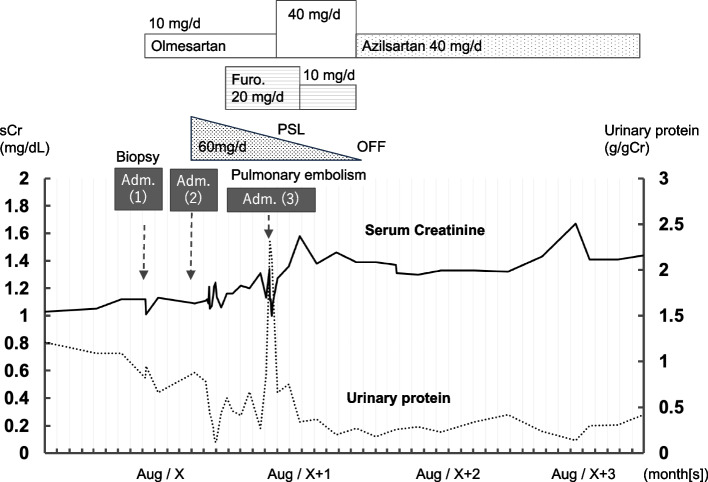
Clinical progression chart. Renal biopsy was performed during the initial hospitalization, followed by the initiation of prednisolone therapy at a dosage of 60 mg (1 mg/kg/day) on subsequent admission. However, renal function did not improved, resulting in a gradual reduction of the steroid dose

FN plays a crucial role in cellular processes such as cell growth, differentiation, migration, and tissue remodeling, including wound healing in response to cellular injury [7]. Therapeutically, steroids acted on inflammatory cytokines and immune cells, making them effective in treating a broad spectrum of diseases. However, steroid therapy is associated with various adverse events, such as hypertension, diabetes, obesity, and increased susceptibility to infections [8]. In the present case, renal function deteriorated during steroid therapy. Although diuretic use may have contributed to this decline, renal function remained worse even after discontinuing both the diuretic and prednisolone therapy (Fig. 3). However, no definitive increase in proteinuria was noted, partly due to the beneficial effects of ARBs presumably. The transient decrease in urinary protein during 3 weeks of hospitalization was likely attributed to the effects of a low-salt diet and bed rest, rather than the direct effect of prednisolone therapy.

The p.(Pro1472del) variant identified in the present case was reported in two Japanese families [5], and the variant was located in the integrin-binding domain. In the literature, a member of family 4 developed end-stage kidney disease in their 40 s, whereas a member of family 10 presented with severe proteinuria, characterized by a UPCR level of approximately 5 g/gCr. By contrast, our case exhibited a relatively milder phenotype. These cases highlight the phenotypic variability associated with the variant and suggest the potential involvement of other modifier genes or environmental factors.

Corticosteroid therapy is commonly attempted in cases with a histological diagnosis of MPGN [9]. A study reported successful treatment in FNG cases with nephrotic-level proteinuria [10]. However, in the present case, we did not observe a clear treatment response, and only adverse events were evident. Steroids are known to enhance the production of coagulation factors and fibrinogen, consequently augmenting blood coagulation capacity and predisposing to thrombus formation [11]. This was considered as a potential side effect in this particular case. Currently, no specific treatment is available for FNG, and angiotensin-converting enzyme inhibitors and ARBs are generally used for renal protection. The initiation of steroid therapy for FNG should be carefully considered on an individual basis.

Although the in-frame deletion in *FN1* identified in this study was previously reported, the accompanying pathological tissue findings were not presented [5]. Therefore, this report presents the first example of histological evidence corresponding to the p.(Pro1472del) mutation, which resulted in MPGN.

In conclusion, FNG must be considered a potential diagnosis in patients presenting with MPGN. MPGN encompasses a broad spectrum of underlying diseases, and patients may have aHUS or other hereditary conditions in the background. However, in adult-onset MPGN with an unclear family history, these possibilities are not actively considered, which can be a pitfall. To investigate underlying factors associated with MPGN, genetic testing may help avoid steroid therapy, which can lead to adverse events. Thus, the treatment approach for each case should be thoroughly evaluated and tailored accordingly.

### Supplementary Information

**Additional file 1: Supplementary Fig. 1. **Immunofluorescence (IF) staining of the renal biopsy. The results of IF staining showed weak deposition of immunoglobulin (Ig) G, with no evident deposition of other IgA, IgM, C3c, C4, or C1q.

**Additional file 2: Supplementary Table.** 121 known genes validated in the targeted NGS panel.

## Data Availability

The data that support the results of this study are available on request from the corresponding author. The data are not publicly available because of privacy or ethical restrictions.
