# Supplementary material for: A case of unexpected diagnosis of fibronectin glomerulopathy with histological features of membranoproliferative glomerulonephritis
Source: BMC Nephrol. 2024 Jan 22;25:25. doi: 10.1186/s12882-024-03456-7 (PMC10802068; doi:10.1186/s12882-024-03456-7)
Supplement: Supplementary file 1 — Additional file 1: Supplementary Fig. 1. Immunofluorescence (IF) staining of the renal biopsy. The results of IF staining showed weak deposition of immunoglobulin (Ig) G, with no evident deposition of other IgA, IgM, C3c, C4, or C1q. [file 12882_2024_3456_MOESM1_ESM.pdf]

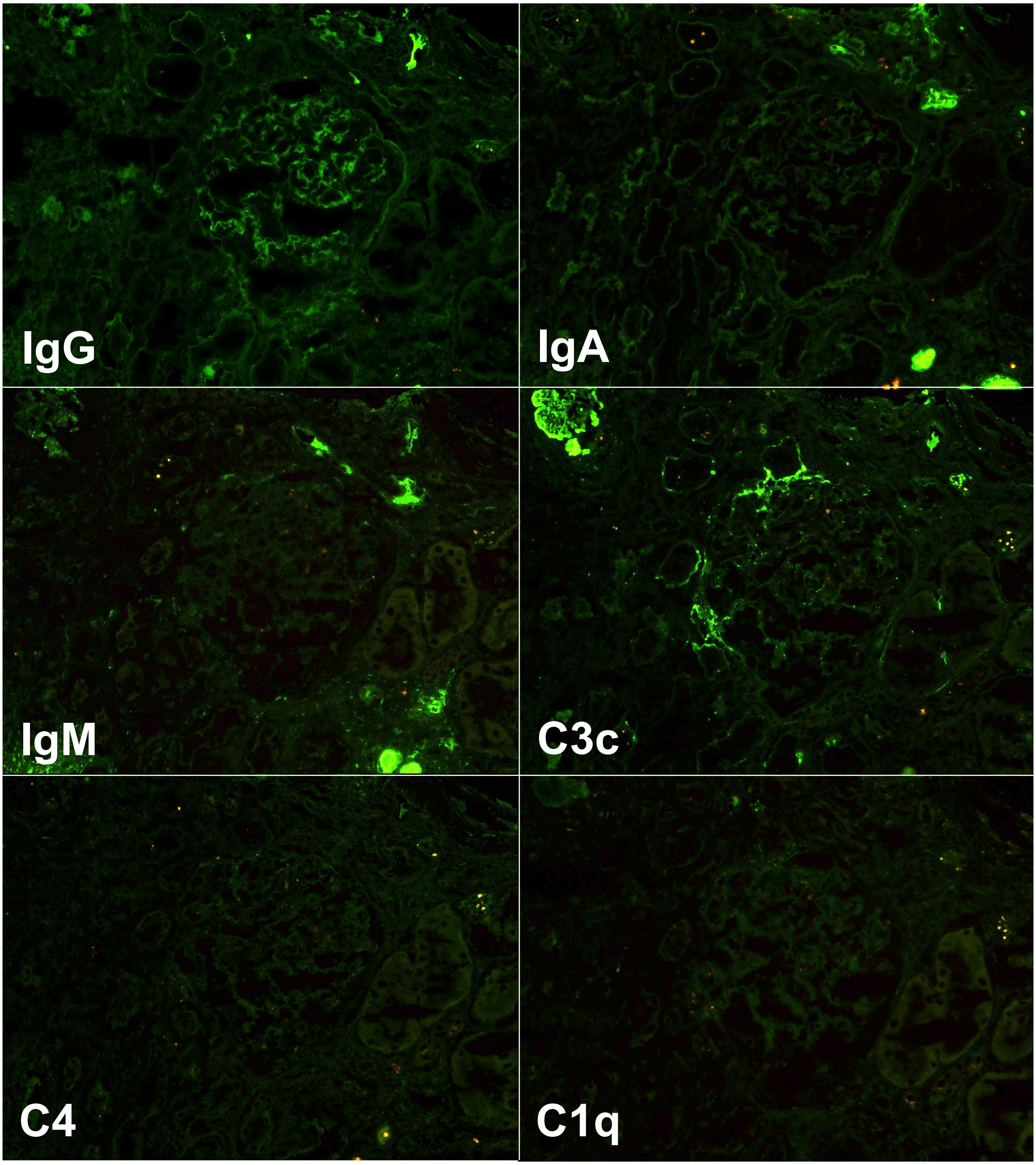

Supplementary Fig. 1
Immunofluorescence (IF) staining of the renal biopsy. The results of IF staining showed weak deposition of immunoglobulin (Ig) G, with no evident deposition of other IgA, IgM, C3c, C4, or C1q.
