# Supplementary material for: A case of unexpected diagnosis of fibronectin glomerulopathy with histological features of membranoproliferative glomerulonephritis
Source: BMC Nephrol. 2024 Jan 22;25:25. doi: 10.1186/s12882-024-03456-7 (PMC10802068; doi:10.1186/s12882-024-03456-7)
Supplement: Supplementary file 2 — Additional file 2: Supplementary Table. 121 known genes validated in the targeted NGS panel. [file 12882_2024_3456_MOESM2_ESM.pdf]

Supplementary Table

121 known genes validated in the targeted NGS panel

ABCG2	*ATP7B*	*CUBN*	*ITGB4*	*NUP205*	*SARS2*
ACE	*C3*	*DGKE*	*ITSN1*	*NUP85*	*SIX1*
ACTN4	*CD2AP*	*DLC1*	*ITSN2*	*NUP93*	*SIX5*
ADAMTS13	*CD46*	*EMP2*	*LAMB2*	*PAX2*	*SLC17A5*
AGL	*CFB*	*EYA1*	*LDLR*	*PCSK9*	*SLC37A4*
AGTR1	*CFH*	*FAT1*	*LMX1B*	*PDSS2*	*SMARCAL1*
ANKFY1	*CFHR1*	*FBN1*	*LPL*	*PFKM*	*STAP1*
ANLN	*CFHR3*	*FGA*	*LYZ*	*PKD1*	*TBC1D8B*
ANOS1	*CFHR5*	*FGFR1*	*MAGI2*	*PKD2*	*THBD*
APOA1	*CFI*	*FN1*	*MEFV*	*PKHD1*	*TNS2*
APOA2	*COL4A3*	*G6PC*	*Mitochondria*	*PLCE1*	*TNXB*
APOA5	*COL4A4*	*GAPVD1*	*MUC1*	*PLG*	*TRPC6*
APOB	*COL4A5*	*GATA3*	*MYH9*	*PODXL*	*TSC1*
APOC3	*COL5A1*	*GLA*	*MYO1E*	*PROK2*	*TSC2*
APOE	*COL5A2*	*GPIHBP1*	*NLRP3*	*PROKR2*	*TTC21B*
APOL1	*COQ2*	*GSN*	*NPHS1*	*PRPS1*	*TTR*
APP	*COQ6*	*HNF1B*	*NPHS2*	*PTPRO*	*UMOD*
APRT	*COQ8B*	*HPRT1*	*NUP107*	*PYGM*	*VHL*
ARHGAP24	*CRB2*	*INF2*	*NUP133*	*REN*	*WT1*
ARHGDIA	*CTNS*	*ITGA3*	*NUP160*	*SALL1*	*XDH*
ASS1					
